# Non-Specific Lipid Transfer Proteins in *Triticum kiharae* Dorof. et Migush.: Identification, Characterization and Expression Profiling in Response to Pathogens and Resistance Inducers

**DOI:** 10.3390/pathogens8040221

**Published:** 2019-11-05

**Authors:** Tatyana I. Odintsova, Marina P. Slezina, Ekaterina A. Istomina, Tatyana V. Korostyleva, Alexey S. Kovtun, Artem S. Kasianov, Larisa A. Shcherbakova, Alexander M. Kudryavtsev

**Affiliations:** 1 Laboratory of Molecular-Genetic Bases of Plant Immunity, Vavilov Institute of General Genetics RAS, 119333 Moscow, Russia; omey@list.ru (M.P.S.); mer06@yandex.ru (E.A.I.); tatkor@vigg.ru (T.V.K.); 2 Phystech School of Biological and Medical Physics, Moscow Institute of Physics and Technology, Dolgoprudny, 141701 Moscow Reg., Russia; kovtunas25@gmail.com; 3 Laboratory of Computational Genetics and System Biology, Vavilov Institute of General Genetics RAS, 119333 Moscow, Russia; artem.kasianov@gmail.com; 4 Laboratory of Plant Genomics, The Institute for Information Transmission Problems RAS, 127051 Moscow, Russia; 5 Laboratory of Physiological Plant Pathology, All-Russian Research Institute of Phytopathology, B. Vyazyomy, 143050 Moscow Reg., Russia; larisavniif@yahoo.com; 6 Laboratory of Plant Genetics, Vavilov Institute of General Genetics RAS, 119333 Moscow, Russia; 2456376@gmail.co

**Keywords:** antimicrobial peptides, non-specific lipid-transfer proteins, transcriptome, induced resistance, *Triticum kiharae*, *Fusarium* spp., elicitor metabolites

## Abstract

Non-specific lipid-transfer proteins (nsLTPs) represent a family of plant antimicrobial peptides (AMPs) implicated in diverse physiological processes. However, their role in induced resistance (IR) triggered by non-pathogenic fungal strains and their metabolites is poorly understood. In this work, using RNA-seq data and our AMP search pipeline, we analyzed the repertoire of nsLTP genes in the wheat *Triticum kiharae* and studied their expression in response to *Fusarium oxysporum* infection and treatment with the intracellular metabolites of *Fusarium sambucinum* FS-94. A total of 243 putative nsLTPs were identified, which were classified into five structural types and characterized. Expression analysis showed that 121 TkLTPs including sets of paralogs with identical mature peptides displayed specific expression patters in response to different treatments pointing to their diverse roles in resistance development. We speculate that upregulated nsLTP genes are involved in protection due to their antimicrobial activity or signaling functions. Furthermore, we discovered that in IR-displaying plants, a vast majority of nsLTP genes were downregulated, suggesting their role as negative regulators of immune mechanisms activated by the FS-94 elicitors. The results obtained add to our knowledge of the role of nsLTPs in IR and provide candidate molecules for genetic engineering of crops to enhance disease resistance.

## 1. Introduction

Non-specific lipid-transfer proteins (nsLTPs) are low-molecular-weight cysteine-rich proteins discovered in all land plants [1,2,3,4,5]. They possess a conserved eight-cysteine motif (ECM): C-Xn-C-Xn-CC-Xn-CXC-Xn-C-Xn-C, with all cysteines being engaged in disulfide bonding. Disulphide bridges stabilize the nsLTP’s three-dimensional structure composed of four or five alpha-helices, connecting loops and unstructured C-terminal region, and render the molecule thermal and chemical stability. Due to a hydrophobic tunnel-like cavity present in lipid-transfer protein (LTP) molecule, these polypeptides are capable of accommodating and transferring different lipid molecules in vitro that suggested a similar role for these polypeptides in vivo. However, further studies showed that LTPs are synthesized as preproteins with a signal peptide targeting them to the apoplast that made this initial suggestion unlikely. nsLTPs are encoded by multigene families with dozens of family members identified by in silico mining and RNA-seq in both cultivated and wild plant species belonging to different families: Poaceae (*Hordeum vulgare, Oryza sativa*, *Zea mays*, *Sorghum bicolor, Leymus arenarius*, and *Triticum aestivum*) [6,7,8,9,10], Brassicaceae (*Arabidopsis thaliana, Brassica rapa*, *Brassica oleraceae*) [6,11,12], Solanaceae (*Solanum esculentum* and *Solanum tuberosum*) [13,14,15], Caryophyllaceae (*Stellaria media*) [16], and Malvaceae (*Gossypium* sp.) [17,18] (Table S1). Several classification systems were suggested for plant nsLTPs that take into account their molecular weight, sequence similarity, the number of amino acid residues between cysteine residues and the position of intron(s) in the corresponding genes [6,19,20,21]. nsLTPs are involved in a wide range of physiological processes, including defense against biotic and abiotic stress, where they act either as antimicrobial agents or signaling molecules, cell expansion, pollen, fruit and seed development and germination, nodule formation, and synthesis of lipid barrier polymers, such as suberin, sporopollenin and cuticular waxes [2,3,4,5]. The participation of nsLTPs in defense against pathogens together with their relatively small molecular size formed the basis for their assignment to antimicrobial peptides (AMPs) and class 14 pathogenesis-related proteins (PR-proteins) [22,23]. Suppression of fungal growth is supposed to be due to an increase in membrane permeability. In addition to important physiological functions mentioned above that make nsLTP genes promising candidates for genetic transformation of plants to increase resistance to pathogenic microorganisms, nsLTPs show potential as templates for drug development in medicine displaying antimicrobial activity against human bacterial pathogens and inhibiting clinical sepsis [24].

Wheat is one of the major cereal crops. Its average annual global production amounted to 757 million tons over the period from 2015 to 2017 (http://www.fao.org/faostat/en/#data/QC). It is the main source of starch and energy for the majority of human population providing 55% of carbohydrates [25]. Wheat also provides compounds essential and/or beneficial for human health such as proteins, vitamins, dietary fiber, and phytochemicals (phenolics and terpenoids) [25]. Dietary fiber is particularly important because it reduces the risk of cardio-vascular diseases, certain forms of cancer and type 2 diabetes. The major wheat species are a hexaploid species *Triticum aestivum* and a tetraploid *Triticum durum* used to make bread and pasta, respectively.

Diseases caused by pathogenic microorganisms lead to considerable production losses and affect quality of agricultural products. Fungal diseases have the greatest impact on wheat production reducing yields by 15–20% [26]. The development of management strategies requires comprehensive knowledge of the plant’s defensive arsenal and the molecular mechanisms of plant–pathogen interactions.

Studies of plant innate immunity showed that endophytic and saprophytic fungi, as well as non-pathogenic fungal strains and their metabolites can protect plants against pathogens by producing toxic secondary metabolites, enzymes and elicitors, which are able to induce systemic resistance of the whole plant to a wide range of pathogenic microbes [27,28]. These compounds are used as biocontrol agents that provide alternative to chemicals and environmentally safe measures of disease management. Although for particular plant–microbe combinations, the interactions between plants and pathogenic and nonpathogenic microorganisms during induced resistance (IR) have been partially clarified, the role of antimicrobial peptides remains poorly understood.

In our previous studies, we demonstrated that the intracellular elicitor metabolites of the biocontrol isolate FS-94 of *Fusarium sambucinum* protect wheat plants from *Stagonospora nodorum,* a causal agent of glume/leaf blotch, and from the fungi of the pathogenic root rot complex (*F. avenaceum, F. culmorum*, *F. sporotrichioides*, *F. oxysporum, F. gibbosum*, and *Bipolaris sorokiniana*) [29,30] and trigger systemic resistance in plants [30,31]. To shed light on the mode of action of the elicitors, we performed global transcriptome sequencing (RNA-seq) of wheat plants treated with the elicitors and expressing IR [32]. A highly resistant to pathogens hexaploid wheat species *Triticum kiharae* Dorof. et Migush., which is a synthetic allopolyploid obtained by crossing *Triticum timopheevii* and *Aegilops tauschii*, was used for transcriptome analysis. Using our earlier developed algorithm for AMP mining in transcriptome data sets, we analyzed the repertoire of nsLTP genes in *T. kiharae* and monitored changes in nsLTP gene expression to identify the genes activated by the *F. sambucinum* elicitor metabolites [32]. In this work, we continue to explore the role of AMPs in the resistance mechanisms induced in *T. kiharae* plants by the *F. sambucinum* elicitor metabolites and focus on nsLTPs, the most abundant AMP family. Using our previously obtained RNA-seq data, we for the first time analyzed the repertoire of putative nsLTPs in *T. kiharae* seedlings and characterized the structure of predicted polypeptides. To elucidate nsLTP functions, we performed expression profiling of nsLTP genes in untreated and elicitor-pretreated wheat in response to infection with the pathogenic *F. oxysporum* strain. Finally, dozens of nsLTP genes involved in *F. oxysporum* resistance development triggered by the FS-94 elicitors were identified in wheat.

## 2. Results

### 2.1. Identification nsLTPs in T. kiharae Transcriptomes and Their Classification

Earlier, we showed that treatment of *T. kiharae* seeds with the elicitor metabolites of *F. sambucinum* (strain FS-94) effectively protects wheat seedlings from *F. oxysporum* infection. To investigate the role of nsLTPs in induced resistance, we first studied the repertoire of nsLTPs in four transcriptomes: from untreated, infected, elicitor-treated, and IR-displaying wheat seedlings. Using two approaches—hidden Markov models and regular expressions, we identified putative nsLTPs in wheat transcriptomes (Table S2).

Initially 260 sequences, which had a stop codon at the end of the precursor protein, were retrieved from the transcriptome dataset. Each sequence was examined for the presence of the ECM typical for LTPs. Seven sequences were discarded because they lacked the signal peptide. Ten sequences of proline-rich proteins with the mature peptide length exceeding 204 residues were also removed. As a result, 243 sequences of putative nsLTPs named TkLTPs remained (Table S2).

All discovered sequences were checked for the presence of the domain characteristic of the lipid-binding protein family (Table S2).

In the precursor proteins, the position of the signal peptide and the presence of the glycosylphosphatidylinositol (GPI) anchor site were predicted. Identified TkLTP precursors differed both in amino acid sequences and the number of residues between the adjacent cysteine residues in the mature peptide region. To classify discovered TkLTP sequences, we used the classification system of Edstam et al. [21]. It should be noted that several classifications of nsLTPs have been developed. Initially they were separated according to molecular weight into two groups LTP1 (molecular weight of 9 kDa) and LTP2 (molecular weight of 7 kDa) [19]. The two groups also differ in disulphide bond pairing. Boutrot et al. [6] classified nsLTPs of *A. thaliana,* rice, and wheat into Types I-IX according to sequence similarity and the number of amino acid residues between the neighboring cysteine residues, with eight nsLTP Types (I-VIII) and 33 subfamilies being discovered in *T. aestivum* (including those with additional cysteines in the ECM motif, and those lacking CXC submotif). Further development of the LTP classification system was the nomenclature suggested by Edstam et al. [21] and elaborated for LTPs of all land plants including not only flowering plants but non-seed land plants as well. It takes into account sequence similarity, the presence of GPI modification site, the number of residues between the adjacent cysteine residues, and the position of intron(s). This classification retained Types 1 and 2 and added subfamilies of Types C-K. These new subfamilies overlap with some of those of Boutrot et al. [3,6].

To classify putative TkLTPs, we first isolated nsLTPs with the GPI modification site in Type G nsLTPs. Sequences without the GPI anchor site after multiple sequence alignments of precursor proteins were separated into groups and classified into Types 1, 2, and D according to the type-specific cysteine spacing patterns suggested by Edstam et al. [21] (Table 1, Figure S1). Types 1 and 2 were the same as Types I and II of Boutrot et al. [6]. In Type D sequences, types IV, V, VI, VIII and XI of Boutrot et al. [6] and Li et al. [12] were further isolated (Table S2). Sequences with novel cysteine spacing patterns were assigned to Type X. The number of peptides in each of five groups (1, 2, D, G and X) was different. The most abundant was Type G group including 91 polypeptides, followed by Type 1 with 59 members.

### 2.2. Validation of TkLTP Gene Expression by RT-PCR

To prove expression of TkLTPs predicted from the RNA-seq data, 40 transcripts taken from different structural types were selected. Wheat total RNA was reverse transcribed, after that PCR with specific primers (Table S3) was carried out. PCR fragments obtained with high-fidelity polymerase were cloned and sequenced. The nucleotide sequences of all 40 selected transcripts were confirmed.

### 2.3. Sequence Analysis of TkLTPs

The characteristics of 243 TkLTPs are presented in Table S2. The length of the predicted signal peptides varied from 13 to 35 amino acid residues. Most TkLTPs were predicted to be secreted, except for TkLTP2.2, which is a chloroplast protein, and TkLTP1.25, TkLTPd7.3, TkLTPg9.1, and TkLTPg10.2 predicted to be located in mitochondria. After cleavage of signal peptides, the mature proteins of several TkLTP precursors appeared identical (colored similarly in Table S2), for example, TkLTP1.2 and TkLTP1.4, suggesting recent gene duplication events as a mechanism of nsLTP gene evolution. Therefore, 243 putative *T. kiharae* nsLTP genes encode 199 different mature LTPs. The molecular weight of predicted mature LTPs except for Type G polypeptides varied from 6976 Da to 14417 Da (the number of amino acid residues varied from 67 to 142). In Type G TkLTPs, the molecular weight of mature nsLTPs was higher because of the presence of the GPI anchor site: from 12961 Da to 20180 Da. The calculated isoelectric points of TkLTPs were from 3.185 to 9.963. The gene ontology (GO) annotations of discovered putative TkLTPs in the category “Biological process” was lipid transport (GO 0006869) and lipid binding in the category “Molecular function” (GO 0008289), and systemic acquired resistance for DIR1-like nsLTPs (GO 0005504) (Table S2).

**Figure 1:**
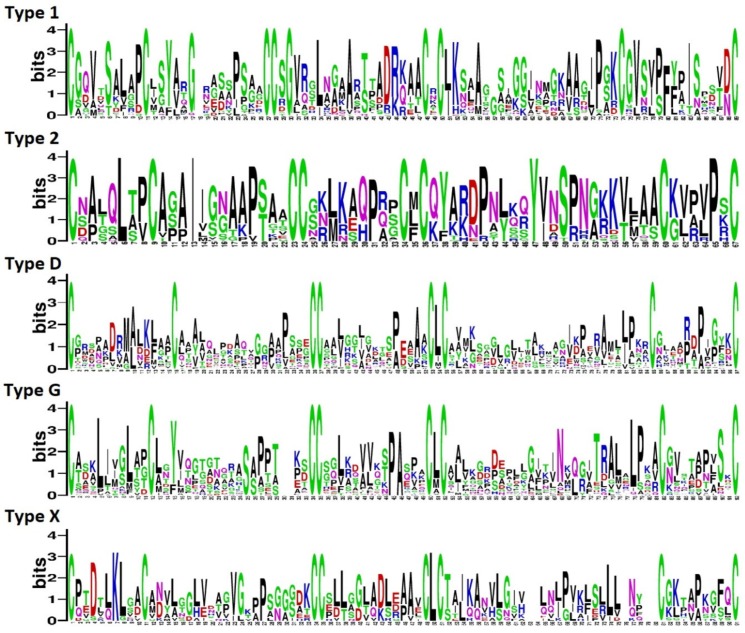
Sequence logo plots of aligned eight-cysteine motif (ECM) sequences for each *Triticum kiharae* lipid-transfer proteins (TkLTP) type. The height of an amino acid residue reflects its conservation level. On the x-axis, the numbers indicate the position of a residue in the ECM. On the y-axis, the information content is shown in bits.

Multiple sequence alignment of TkLTP precursor proteins revealed groups (subfamilies) of related polypeptides in each structural type characterized by the conserved ECM (Figure S1). ECM sequence Logos were constructed for each TkLTP type to explore the conservation of amino acid residues between the adjacent cysteine residues (Figure 1). The degree of conservation was the highest in Types 2 and 1, and the lowest, in Types D and G.

Sequence analysis showed that in the CXC motif, which is supposed to influence the cysteine pairing and fold of the LTP molecule [11], a hydrophobic residue usually occurs in Type 2 nsLTPs and a hydrophilic residue in Type 1 nsLTP sequences, as for example, in cabbage [11] and maize [8]. In putative TkLTPs, twelve different residues were found in the CXC motif (R, T, K, N, L, G, E, A, M, F, V, I). In Types 2, D and G, only hydrophobic residues (L, M, F, V, I, A) were located in this position, with L being found in the majority of sequences. In Type 1 TkLTPs, R, T, K, N, L, G, E, A were present in the CXC motif. The same residues except for E were discovered in this position in *T. aestivum* nsLTPs by Boutrot et al. [6].

In *T. kiharae* putative nsLTP sequences, of two conserved pentapeptides T/SXXDR/K and PYXIS, which are supposed to be crucial for lipid binding or catalysis [33], 49 sequences harbored the pentapeptide T/SXXDR/K, and 27 sequences possessed also the PYXIS motif. Thus, the former pentapeptide is more conserved than the latter one. The same observation was made for *B. oleraceae* nsLTPs [11].

Discovered putative *T. kiharae* nsLTP precursors showed the highest sequence similarity to nsLTPs (192 proteins), proline-rich proteins (7) and unnamed proteins (44) from hexaploid wheat genome donors *Ae. tauschii* (subgenome D, 165 proteins) and *T. urartu* (subgenome A, 12 proteins), the bread wheat *T. aestivum* (AABBDD, 56 proteins), *Brachypodium distachyon* (four proteins), and *H. vulgare* (six proteins) (Table S2). Nine TkLTPs showed sequence similarity to the “defective in induced resistance” (DIR1) protein of *A. thaliana* involved in signal transduction during systemic acquired resistance (SAR). *A. thaliana* DIR1 belongs to a specific group of acidic nsLTPs of the LTP2 family possessing a PXXPXXP motif supposed to be involved in interactions with various protein domains [34]. In *T. kiharae,* not all DIR1 orthologs are acidic proteins (TkLTPd6.1–d6.4 are neutral), and their proline motifs are different from those of *A. thaliana* and in various structural types: in TkLTPd3, PXXPPXXX; in TkLTPd6, PXXXPXXX; and in TkLTPd7, PXPXPXXX.

**Figure 2:**
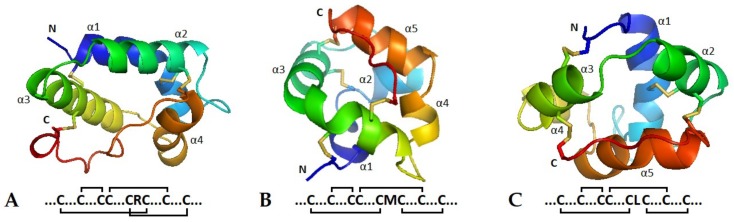
Three-dimensional structure and cysteine-pairing pattern of TkLTP1.36 (**A**), TkLTP2.21 (**B**) and TkLTPd7.1 (**C**). Disulfide bonds are shown by yellow color.

In order to shed light on the three-dimensional structure of TkLTPs, three sequences of TkLTP1.36, TkLTP2.21 and TkLTPd7.1 were chosen for molecular modeling (Figure 2). All three *T. kiharae* polypeptides possess the typical for nsLTPs tertiary structure with four in Type 1 and five in Types 2 and D α-helical regions connected by loops and surrounding a hydrophobic cavity. However, the cysteine pairing differs in TkLTP1.36 from that of TkLTP2.21 and TkLTPd7.1 (Figure 2) that is possibly associated with a hydrophilic residue R in the CXC motif in Type 1 TkLTP and hydrophobic residues M in TkLTP2.21 and L in TkLTPd7.1 in this motif.

### 2.4. Phylogenetic Analysis

**Figure 3:**
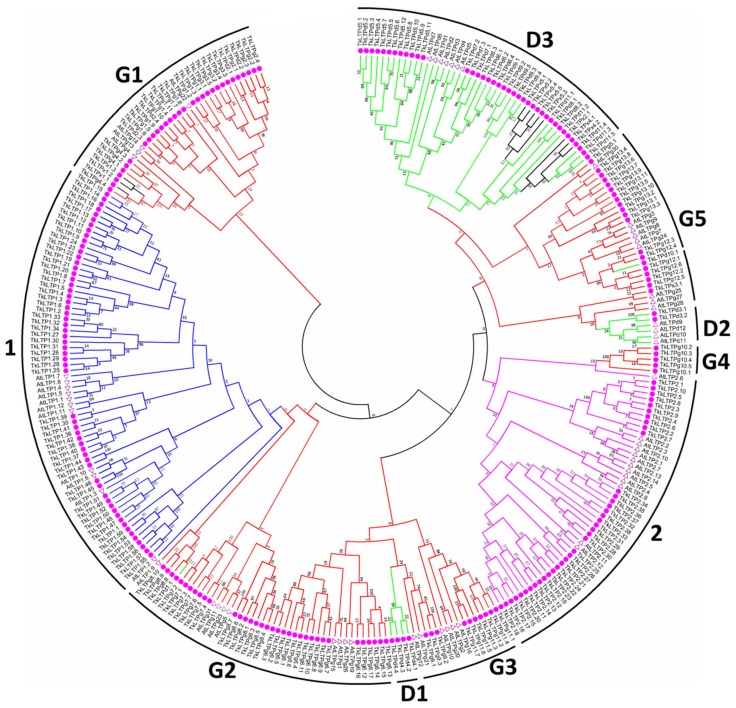
A phylogenetic tree of the putative nsLTPs from *T. kiharae* and *A. thaliana*. The amino acid sequences of the mature nsLTPs were used for the phylogenetic tree construction with the MEGA7 software. *T. kiharae* nsLTPs are marked with pink circles, those of *A. thaliana*—with triangles. The accession numbers of the *A. thaliana nsLTPs* are shown in Table S4. nsLTPs Types are indicated by branches of different colors: Type 1—blue, Type 2—pink, Type D—green, Type G—red, and Type X—black. Bootstrapping was carried out 10,000 times to get support values for each branch.

A phylogenetic tree based on TkLTP and *A. thaliana* mature peptide sequences was constructed by the Neighbor-joining method (Figure 3). The branching of the tree is consistent in general with classification into the main structural types indicating origin from a common ancestor of nsLTP genes within each clade. Types 1 and 2 nsLTPs form distinct clusters, while Type D sequences are subdivided into three, and Type G, into five subclusters. Several Type X sequences are clustered together with Type D3 and Type G1 sequences, and one Type D sequence is in the Type G5 cluster, suggesting that these sequences might have arisen from Type D3, Type G1 and Type G5 genes, respectively.

### 2.5. Expression Analysis of nsLTP Genes

**Figure 4:**
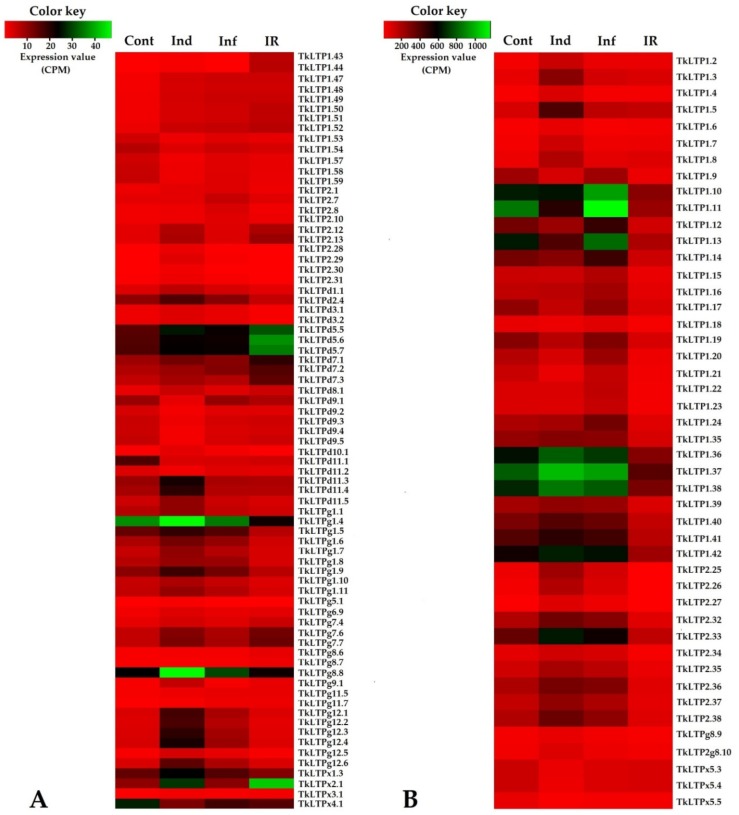
Heatmaps of differentially expressed TkLTP genes. (**A**) Genes with expression levels below 50 CPM in all transcriptomes. (**B**) Genes, whose expression levels were above 50 CPM at least in one transcriptome. The designations are as follows: Cont, Ind, Inf and IR, control, induced, infected and IR-expressing seedlings, respectively.

**Figure 5:**
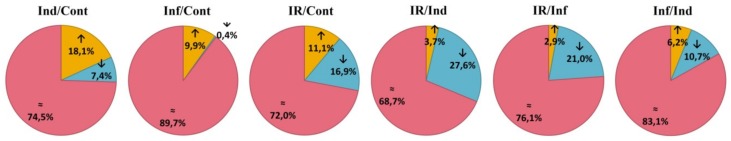
Differentially expressed TkLTP genes in *T. kiharae* transcriptomes (presented in % of the total number of expressed TkLTP genes). Upregulated genes (expression fold change ≥ 2) are shown in orange; downregulated TkLTP genes (expression fold change ≤ 0.5) are given in blue; TkLTP genes whose expression level did not change are colored pink. Above the figures, the designations are as follows: Ind/Cont, elicitor-treated versus control; Inf/Cont, infected versus control, IR/Cont, IR-displaying versus control; IR/Ind, IR-expressing versus elicitor-treated; IR/Inf, IR-expressing versus *F. oxysporum*-infected; Inf/Ind, infected versus elicitor-treated.

We used earlier obtained RNA-seq data [32] to estimate the expression levels of putative TkLTP genes in different transcriptomes and build a heatmap (Table S5, Figure 4). Comparison across all four transcriptomes demonstrated that the expression level of 122 genes did not change more than two-fold and less than 0.5-fold under all treatments (infection, induction, induction + infection). Each treatment altered expression of 10% to 30% genes (Figure 5). Since expression levels differed between family members and in different transcriptomes, we separated TkLTPs into two groups—highly expressed (above 50 CPM at least in one trancriptome) and weakly expressed genes (below 50 CPM in all transcriptomes). The first group included 46 family members, most of which belonged to Types 1 and 2 with only two Type G and three Type X sequences. No Type D nsLTPs were discovered in this group. The weakly expressed group included 75 TkLTP genes encompassing all structural types. Expression profiling showed that even several identical TkLTPs derived from different precursor proteins differed in expression patterns (see for example TkLTP1.9 and TkLTP1.10; 1.22 and 1.24; g11.6 and g11.7 in Figure 4, Table S5).

#### 2.5.1. Infection with Fusarium oxysporum (Inf/Cont)

Infection with *F. oxysporum* changed expression of 10% TkLTP genes, the majority of which (24) were upregulated, and only one TkLTPd11.1 gene was downregulated (Figure 5, Table S5).

Expression level of upregulated TkLTP genes altered from 2.04-fold for TkLTPg11.5 to 3.63 for Tk-LTP1.47 (Table S5). The upregulated genes belonged to Types 1 (six genes), 2 (eight genes), D (1 gene), G (eight genes) and X (one gene), while the downregulated TkLTPd11.1, which showed the highest sequence similarity to a 14 kDa proline-rich membrane protein, belonged to Type D (Tables S5 and S6). It is of interest that all Type 1 upregulated TkLTP genes encode acidic proteins (PI 3,185 and 3,203), while the upregulated proteins of other structural Types include basic, neutral and acidic polypeptides (Table S2).

#### 2.5.2. Treatment with FS-94 Elicitors (Ind/Cont)

Treatment with the FS-94 elicitors changed expression of more TkLTP genes (25%) than infection with *F. oxysporum*. Similarly to infection, more genes were upregulated than downregulated: 18% (44) and 7% (18), respectively (Figure 5). Expression level of upregulated genes changed from 2,0-fold in TkLTPg8.7 to 10.39-fold in TkLTP2.28, while expression level of downregulated genes—from 2,1-fold in TkLTPx4.1 to 4,76-fold in TkLTPd11.1. Upregulated genes belonged to all discovered structural Types: 1 (15 genes), 2 (nine genes), D (five genes), G (13 genes) and X (two genes), and downregulated, to Types 1 (seven genes), D (seven genes) and X (four genes) (Tables S5 and S6). No Types 2 and Type G TkLTP genes were downregulated by the elicitors.

#### 2.5.3. IR-Displaying Plants (IR/Cont)

In *T. kiharae* plants pretreated with the elicitors and subsequently infected with *F. oxysporum*, expression of 28% genes changed compared to control, with more TkLTP genes being downregulated than upregulated: 17% (41) and 11% (27) genes, respectively (Figure 5, Table S6). The upregulated genes included eight Type 1 genes, two Type 2 genes, seven Type D, eight Type G and two Type X genes. Among downregulated genes, the majority belonged to Type 1 (27 genes of all 59 discovered Type 1 genes) followed by Type 2 (10 genes), Type D (three genes), and one Type G gene (Table S6).

#### 2.5.4. Comparison of Treatments

**Figure 6:**
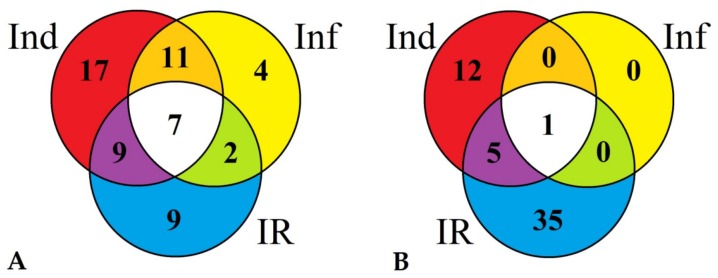
Venn diagram demonstrating the number of TkLTP genes specifically up- or downregulated (in comparison with control) in elicitor-treated (Ind), *F. oxysporum*-infected (Inf) and IR-displaying *T. kiharae* seedlings as well as similarly expressed TkLTPs in all three transcriptomes. (**A**) Upregulated TkLTP genes. (**B**) Downregulated TkLTP genes. For upregulated TkLTP genes, expression fold change was ≥2, for downregulated, ≤0.5.

Comparison of differentially expressed TkLTP genes in infected (Inf/Cont), elicitor-treated (Ind/Cont) and IR-displaying (IR/Cont) wheat seedlings revealed that they represent overlapping groups (Figure 6, Table S6).

Some TkLTP genes were up- or downregulated in all three transcriptomes, and some, in two transcriptomes. Furthermore, some TkLTP genes were specifically up- or downregulated only in one transcriptome. For the upregulated genes, seven genes encoding TkLTP1.47–1.52 and TkLTPx3.1 were upregulated in all instances (Figure 6A, Table S6). Four genes were specifically induced by *F. oxysporum* infection, 17 genes were activated by the elicitors and nine TkLTP genes were upregulated in IR-displaying plants (Figure 6A, Table S6). Of the downregulated genes, only one TkLTPd11.1 gene was downregulated in all three transcriptomes. Thirty-five TkLTP genes were specifically repressed by the elicitors and 12 by infection of elicitor-pretreated plants (Figure 6B, Table S6).

**Figure 7:**
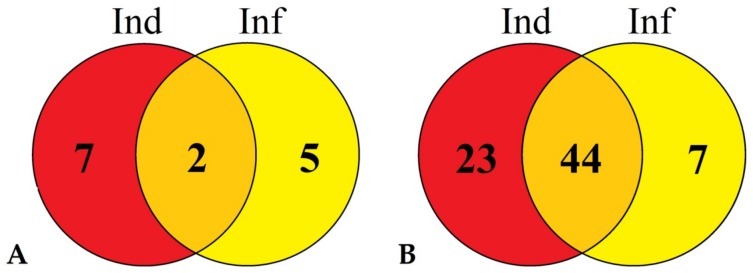
Venn diagram demonstrating the number of TkLTP genes specifically up- or downregulated in IR-displaying seedlings compared with elicitor-treated (Ind) and *F. oxysporum*-infected (Inf) seedlings as well as similarly expressed TkLTP genes in both transcriptomes. (**A**) Upregulated TkLTP genes. (**B**) Downregulated TkLTP genes. For the upregulated TkLTP genes, expression fold change was ≥2, for downregulated, ≤0.5.

Comparison of IR-displaying (treated with the elicitors and infected) plants with infected plants with disease symptoms (IR/Inf) showed that the majority of TkLTP genes were downregulated 21% (51), and only 3% (7) genes were upregulated (Figure 7, Table S7). The upregulated genes included two Type 1 genes (TkLTP1.43 and TkLTP1.44), two Type 2 genes (TkLTP2.12.and TkLTP2.13), one Type D gene (TkLTPd7.3), one Type G gene (TkLTPg9.1) and one Type X gene (TkLTPx2.1) (Table S7). TkLTP1.43 and TkLTP1.44 were similar to a putative neutral lipid-transfer protein of *Ae. tauschii* (EMT27039.1) (Table S2). TkLTP2.12 and TkLTP2.13 displayed similarity at the amino acid level to the unnamed protein product of *T. aestivum* (CDM81734.1). TkLTPd7.3 showed the highest sequence similarity to a putative lipid-transfer protein DIR1 of *T. urartu* (EMS50543.1). TkLTPg9.1 and TkLTPx2.1 were similar to two *H. vulgare* predicted proteins (BAK08128.1 and BAJ97873.1, respectively).

The downregulated genes belonged to all identified TkLTP Types. Among them, two acidic DIR1 orthologs TkLTPd3.1. and d3.2, several YLS3-like nsLTPs (TkLTPg1.5, TkLTPg1.7 and g1.8) and VAS LTPs (TkLTPd10.1, TkLTPg12.1–g12.6) were found. In *A. thaliana*, VAS (Vascular Tissue Size) gene is involved in plant development influencing vascular cell number [35], and YLS (Yellow Leaf Specific) genes are upregulated during normal senescence (17) and are connected, at least in some species, with drought tolerance [36].

Comparison of IR-displaying (treated with the elicitors and infected) plants with those treated with the elicitors (IR/Ind) also showed the prevalence of downregulated TkLTP genes over upregulated ones (27% versus 4%) (Table S7, Figure 7). Only nine genes were upregulated. Of them, TkLTP1.43 and TkLTP1.44 were also upregulated in infected plants. Of the downregulated genes (67), the vast majority was also downregulated by the elicitor treatment (44) including the abovementioned orthologs of *A. thaliana* DIR1, VAS and YLS nsLTPs (Table S7).

Thus, TkLTPs expression analysis showed that most variation in TkLTP gene expression levels was observed in Type 1 (Figure 4).

### 2.6. Validation of RNA-seq-Based Expression Patterns by qRT-PCR

**Figure 8:**
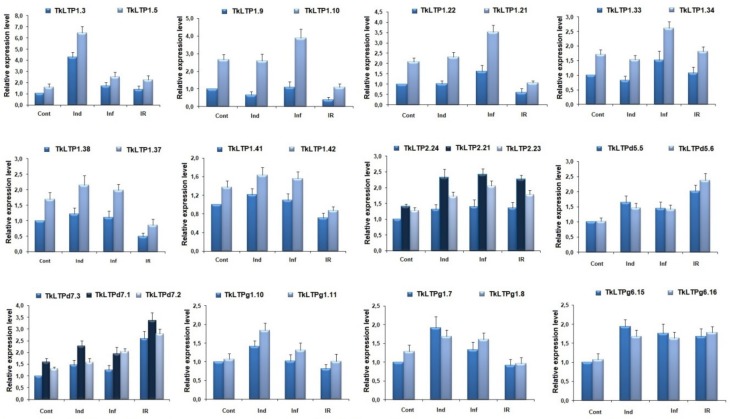
qRT-PCR validation of expression levels for selected TkLTP genes. Relative expression values were normalized using the *rli* gene as internal control and standardized relative to the control values. Analyses were accomplished in triplicate; bars represent standard deviation.

To prove the expression profiles of TkLTP genes generated by RNA-seq, we used qRT-PCR. 26 genes encoding groups of paralogs with identical mature peptides were chosen for analysis. The *rli* gene was used as an internal control since it was more stable under different treatments in wheat. Our qRT-PCR results confirmed that the expression patterns of all selected genes were consistent with the RNA-seq data (Figure 8).

## 3. Discussion

Although the 3D solution structure of several *T. aestivum* nsLTPs unassociated with a ligand and in complexes with different ligands has been resolved either by NMR spectroscopy or by X-ray crystallography nearly twenty years ago [37,38,39,40], the functions of the vast majority of the family members and their role in disease resistance remain poorly understood. Most available data point to positive regulation of disease resistance by wheat nsLTPs. This follows from in vitro assays of antimicrobial activity and analysis of transgenic plants overexpessing wheat nsLTP genes. Thus, in vitro antifungal assays with eight *T. aestivum* nsLTPs against eight wheat and three non-wheat pathogens carried out by Sun and coauthors [41] showed differential inhibition of mycelium growth or spore germination. It is noteworthy that among the antifungal LTPs studied by Sun et al. [41], two polypeptides were 100% identical to TkLTP1.17 and TkLTP1.21, and two LTPs showed high sequence similarity (98.8% and 96.6%) to TkLTP1.15 and TkLTP1.16, respectively. All tested *T. aestivum* LTPs displayed in vitro activity against *F. graminearum*, the TkLTP1.15 ortholog was especially potent [41]. However, none of *T. kiharae* nsLTPs orthologs appeared to be upregulated by *F. oxysporum* infection, or *F. sambucinum* elicitors in our experiments (see below) pointing to the specificity of the immune response to *Fusarium* species. In vitro studies of the biological activity of wheat nsLTPs in a human model using umbilical vein endothelial cells (HUVEC) demonstrated antioxidant and cytoprotective activities of nsLTP2 through decreasing the level of reactive oxygen species (ROS) [42]. The *T. kiharae* ortholog TkLTP2.34 (100% identical to the *T. aestivum* polypeptide) was nevertheless downregulated in IR-displaying *T. kiharae* plants (see below) indicating that either mounting of defense response by the FS-94 elicitors is not associated with inhibition of ROS production, or this function is accomplished by other upregulated TkLTPs or even other proteins (non-LTPs). The available literature data indicate that expression of some *T. aestivum* nsLTPs is upregulated by microbial infection and insect pests [43,44]; the expression level is usually higher in resistant varieties than in susceptible ones. However, Kürkcüoglu et al. [45] observed downregulation of a wheat 9 kDa non-specific lipid transfer protein in the apoplast of *Malus domestica* after the application of the non-pathogenic bacterium *Pseudomonas fluorescens* Bk3 to the leaves.

In several studies, overexpression of wheat LTP genes in transgenic plants was shown to enhance pathogen resistance. For example, Zhu et al. [43] reported that overexpression of the wheat lipid transfer protein TaLTP5 gene in transgenic wheat increased resistance to two important wheat pathogens, *Cochliobolus sativus* and *F. graminearum* causing head blight and common root rot, respectively. Transgenic *A. thaliana* plants expressing the TdLTP4 gene from *T. turgidum* showed increased fungal resistance against *Botrytis cinerea* and *Alternaria solani* [46]. Furthermore, combined expression of a wheat lipid transfer protein and a barley chitinase chi-2 in transgenic carrot plants increased resistance to *Alternaria radicicola* and *B. cinerea*.

In addition to pathogen resistance, association with abiotic stress tolerance was demonstrated for several wheat LTPs. Firstly, several studies showed that wheat LTPs are induced by abiotic stressful factors. Thus, Hairat et al. [47] reported that two wheat LTPs, TaLTP40 and TaLTP75, were highly expressed during cold, drought and salt stress. Secondly, transgenics overexpressing wheat LTP genes exhibit higher tolerance to abiotic stress. Thus, transgenic *A. thaliana* plants overexpressing the TaLTP40 and TaLTP75 genes displayed increased salt tolerance [47]. Three Type I TaLTPs were found to elevate chilling tolerance in transgenic *A. thaliana* [48].

In a recent study, negative regulation of wheat resistance to *Puccinia striiformis* f. sp. *tritici* by TaDIR1-2, which is a wheat ortholog of the *A. thaliana* lipid transfer protein AtDIR1, was demonstrated [49]. The level of the TaDIR1-2 transcript was significantly increased during the compatible interaction with the stripe rust pathogen *P. striiformis*. Knocking down TaDIR1-2 gene expression by virus-induced gene silencing increased resistance of the wheat plants to the pathogen. Enhanced resistance was accompanied by hypersensitive response, elevated levels of H_2_O_2_ and salicylic acid (SA), and suppressed expression of two ROS scavenging genes. The authors suggest that TaDIR1-2 plays a role of a negative regulator in wheat resistance to *P. striiformis* via modulating ROS and/or SA signaling. It deserves special attention that negative regulation of disease resistance by an LTP gene was shown by Gao et al. [50] for a pathogen and abscisic acid (ABA)-induced LTP3 gene of *A. thaliana*. It is of particular interest that this LTP is 53% identical to the *T. kiharae* TkLTP1.38. The overexpression of LTP3 (LTP3-OX) gene enhanced susceptibility to the virulent bacterium *Pseudomonas syringae* pv. *tomato* [50]. Upon infection of the transgenic LTP3-OX plants with *P. syringae*, the genes involved in ABA biosynthesis (NCED3 and AAO3), were highly expressed, meanwhile, the SA-related genes, PR1 and ICS1, were downregulated. In LTP3-OX plants, ABA levels were increased and SA levels were decreased relative to the wild-type plants. The authors suggest that LTP3 is a negative regulator of plant immunity which acts via the perturbation of the ABA-SA balance. In our experiments, the TkLTP1.38 gene was also downregulated in IR-displaying plants, suggesting a similar function of this gene to the *A. thaliana* ortholog LTP3.

The objective of our work was to shed light on the role of nsLTPs in *F. oxysporum*—*T. kiharae* interactions and in the resistance mechanisms induced by the elicitor metabolites of *F. sambucinum*. Using previously obtained RNA-seq data, we performed comprehensive analysis of *T. kiharae* nsLTP genes and their expression in response to *F. oxysporum* infection, treatment with IR-inducing elicitor metabolites from *F. sambucinum* and in IR-expressing plants.

Using two approaches, hidden Markov models and regular expressions we identified 243 putative TkLTP genes in *T. kiharae* transcriptomes. Thus, nsLTP genes in *T. kiharae* form a multigene family. Genome-wide analysis of other plant species also demonstrates that nsLTPs are represented by multigene families with dozens of family members (Table S1). It is worth noting however, that variation in LTP number is partly due to the source of data used for LTP mining (genome or transcriptome) and different criteria applied for attribution of a sequence to the LTP family. For example, in some instances, sequences with the GPI anchor were included in the family (e.g., [10]), while sometimes they were excluded [6]. Proteins targeting to chloroplasts or mitochondria, proline-rich or hybrid proline-rich proteins were often excluded from the putative nsLTPs (e.g., [14]). Nevertheless, the number of nsLTP genes present in the wheat species *T. kiharae* and *T. aestivum* obviously exceeds that of other plant species. The expansion of nsLTP family is due to the hexaploid nature of both species originated by spontaneous two-step hybridization of the diploids —donors of subgenomes A, B/G and D and to gene duplication as a major mechanism in the Poaceae nsLTP gene evolution [51]. The discovered TkLTPs were classified into five structural Types 1, 2, D, G and X (with a novel cysteine-spacing pattern). IN *T. kiharae*, the most abundant was Type G. It is noteworthy that genome-wide analysis of *T. aestivum* LTPs (cv. Chinese Spring) showed the prevalence of Type 2 sequences over all other Types [10]. It should be noted that in *T. aestivum*, eight nsLTP Types (I-VIII) were distinguished by Boutrot et al. by EST data mining [6]. In *T. kiharae* transcriptomes, Types III and VII were not discovered, while several LTPs with novel cysteine spacing patterns were identified (Table S2).

### 3.1. Infection by the Pathogen

We showed that infection with the pathogenic *F. oxysporum* strain (compatible interaction) affected expression of 10% TkLTP genes, all of them, except one, were upregulated. We assume that upregulated TkLTP genes participate in defense against the pathogen. We showed that they belong to all discovered Types (1, 2, D, G and X) with the prevalence of Type G and Type 2 TkLTPs. All Type 1 LTPs (TkLTP1.47–1.52) represent a group of highly similar polypeptides: TkLTP1.47 and TkLTP1.48 have identical mature peptides, while the remaining sequences (TkLTP1.49–1.52) differ in single amino acid residues. This proves recent origin of these genes by duplication event(s). However, the functions of these genes remain unknown and require further studies. The acidic nature of these polypeptides argues against direct interaction with negatively charged fungal membranes. In accordance with this, they are predicted to be non-AMPs, suggesting their regulatory role. It is of interest that this group of genes is responsive to all treatments being upregulated by infection, elicitor metabolites, and infection after elicitor application (Table S6).

In contrast to Type 1 TkLTPs, upregulated Type 2 genes are likely to exhibit antimicrobial activity according to the AMP predictor. We may speculate that these polypeptides target the pathogen (*F. oxysporum*) directly. However, expression of these genes changes 2–2.5-fold upon the fungal infection, possibly insufficient to suppress disease symptoms.

Most of the remaining upregulated Types D and G TkLTPs, except for TkLTPg11.5 and TkLTPg11.7, are predicted to be non-AMPs. They show the highest sequence similarity to the lipid-transfer protein VAS of *Ae. tauschii* (XP_020157978.1). In *A. thaliana* the VAS gene is expressed in vascular tissues and is involved in the control of the number of phloem (pro)cambial and pericycle cells [35]. We may speculate that activation of VAS nsLTP genes in *T. kiharae* upon infection with *F. oxysporum* is related to the reinforcement of physical barriers to restrict fungal growth. Another possibility is that VAS transfers a lipophilic compound to the apoplast, which acts as a signal affecting vascular tissue growth in infected plants [35].

### 3.2. Treatment with FS-94 Elicitors

Treatment with the FS-94 elicitors changed expression of much more TkLTP genes than *F. oxysporum* infection (25% instead of 10% in infected plants) and again more genes were upregulated than downregulated. Upregulated nsLTP genes belonged to all structural Types. A number of genes upregulated by the elicitors were the same as induced by the *Fusarium* infection. They included in addition to TkLTP1.47–1.52 mentioned above, TkLTP2.25–2.28, TkLTPd10.1, TkLTPg12.1–g12.6, and TkLTPx3.1. However, a set of 17 genes were specifically induced by the elicitors; among them, TkLTP1.2–1.8, 2.29–2.31, d11.3–d11.5, g5.1, g6.9, g8.8, g8.10. The vast majority of elicitor-induced TkLTP genes were annotated by BLAST as lipid-transfer proteins, a few of them, as “uncharacterized” or “unnamed” proteins. Most of them are predicted to exhibit antimicrobial activity; however, some of them are possibly non-AMPs. It is noteworthy that treatment with the elicitors upregulated a considerable portion of Type G TkLTPs. Type G nsLTPs carry a C-terminal signal sequence, to which a GPI-anchor is added post-translationally. This anchor binds proteins to the extracellular side of the plasma membrane. Type G nsLTPs were shown to be involved in the accumulation of cuticular wax, suberin and sporopollenin [52]. We hypothesize that their role in *T. kiharae* may be in strengthening plant cell walls to limit fungal growth. It deserves special attention that in addition to the upregulated TkLTP genes, expression of a set of 18 genes was suppressed by the FS-94 elicitors. They are restricted to only three structural Types 1, D, and X. We may suggest that the repressed genes may either be not involved in resistance development or they act as negative regulators of the immune processes.

### 3.3. Treatment with FS-94 Elicitors Followed by F. oxysporum Infection

In contrast to single treatments (infection or elicitors), in *T. kiharae* seedlings treated with FS-94 elicitors and subsequently infected by *F. oxysporum,* expression of more genes was downregulated than upregulated (41 versus 27). Among upregulated genes, there were those induced by the elicitors (9). Two genes for TkLTPg11.5 and g11.7 were activated by *F. oxysporum* infection. The most interesting fact is that among the upregulated genes, a group of nine genes was not induced by the elicitors or infection alone (TkLTPd5.5–d5.7, d7.1–d7.3, g7.4, g8.6, g8.7). We assume that they were primed by the elicitors to provide a more rapid and efficient defense response against *F. oxysporum* infection. It is of particular interest that in this set of genes, three orthologs of DIR1-like proteins of *A. thaliana,* TkLTPd7.1, TkLTPd7.2 and TkLTPd7.3, were discovered. In *A. thaliana*, DIR1 has been suggested to transport a small signaling molecule down the leaf petiole to distant tissues during SAR [53]. DIR1 orthologs presumably having similar functions were discovered in other plant species, such as *N. tabacum,* tomato, barley, cucumber, soybean and wheat [49,54,55,56,57,58]. It was shown that in *A. thaliana*, DIR1 expression decreased during SAR induction (inoculation of leaves with SAR-inducing avirulent or virulent *P. syringae* pv *tomato* strains) [53]. In our work, we showed that in *T. kiharae,* three DIR1 orthologs, TkLTPd7.1, TkLTPd7.2 and TkLTPd7.3, were upregulated in IR-displaying plants compared to control. DIR1 ortholog TkLTPd7.3 was also upregulated in IR-expressing plants compared to infected seedlings (Table S5). At the same time, other *T. kiharae* DIR1 orthologs—TkLTPd3.1 and TkLTPd3.2, were downregulated, while expression of DIR1 orthologs TkLTPd6.1–d6.4 was unaffected by either treatment (infection, elicitors, infection of elicitor-treated plants) in *T. kiharae*. The discovery of different expression regulation of DIR1 orthologs in *T. kiharae* points to their different role in the resistance mechanisms. This fact is not surprising taking into account low sequence similarity between different structural groups (TkLTPd3, d6 and d7) (Table S2, Figure S1). However, the Logo plot of all aligned *T. kiharae* DIR1 mature peptide sequences (Figure S2) revealed several conserved features in their molecules. Except for conserved eight-cysteine motif, the following conserved motifs and residues were discovered: PXXXPS, Y54, I67, WL, and GXXXP. Conserved cysteine residues were shown to be important for the formation of the hydrophobic cavity [58]. Proline-rich region is supposed to participate in protein-protein interactions [34]. Polar residues in the N-terminal region of the DIR1 molecules at the entrance of the hydrophobic cavity are suggested to stabilize interactions with the putative hydrophilic regions of ligands [34]. A hydrophobic residue between the two cysteines is hypothesized to play an important role in cysteine bond pairing, determination of the size and shape of the hydrophobic tunnel, and interactions with the ligands [58,59]. The role of other conserved residues and motifs in *T. kiharae* DIR1 orthologs and sequence variation underlying different functions is still to be elucidated.

Comparison of IR-expressing *T. kiharae* seedlings with *F. oxysporum*-infected seedlings showed that only 7 putative nsLTP genes were upregulated: 1 DIR1 homolog TkLTPd7.3, 2 putative nsLTPs and 4 predicted proteins with unknown functions (Table S7). We suppose that four of them predicted to be AMPs act as antimicrobial agents, while the remaining three as positive regulators of the immune response. We may speculate that similarly to *A. thaliana* LTPs 4.4 and 4.5, some induced TkLTPs might increase resistance to trichothecene mycotoxins produced by *Fusarium* species through mounting antioxidant defense [60].

Comparison of IR-displaying plants with *F. oxysporum*-infected and elicitor-treated seedlings disclosed an amazing fact that in contrast to the DIR1 ortholog TkLTPd7.3, the vast majority of TkLTP genes including DIR1 orthologs TkLTPd3.1 and d3.2, were downregulated suggesting their role as negative regulators of the induced resistance mechanism. However, detailed functional studies including knock-out mutants are necessary to make definite conclusions regarding their in vivo role.

## 4. Materials and Methods 

### 4.1. RNA-seq Data

Experimental design was described in detail earlier [32]. In short, wheat seeds were immersed in 0.5% KMnO_4_, washed thoroughly with sterile distilled water and incubated at 20–22 °C for 16 h, after that seeds were divided into two groups (100 seeds in each), placed on sterile paper filters in Petri dishes (25 seeds per dish) and treated with sterilized *F. sambucinum* metabolites (50 μL per seed) or distilled water for 2.5–3 h under aseptic conditions. One half of elicitor- and H_2_O-treated seeds were inoculated with *F. oxysporum* strain 137-spore suspension (10^6^ spores/mL, 100 μL per seed). Non-inoculated H_2_O-treated seeds were used as control. After the treatments, 200 germinated seeds were grown for three days at 20–22 °C (the first day in the dark, and then two days under long-day conditions (16 h day/8 h night)), harvested, immediately frozen in liquid nitrogen and stored at −80 °C until total RNA isolation. Thus, four samples of young wheat seedlings were obtained for cDNA library construction and RNA-seq: (1) control group: seeds were germinated in sterile water; (2) induced sample: seeds were germinated in elicitor metabolites of *F. sambucinum* isolate FS-94; (3) infected sample: seeds were germinated in sterile water and further infected with *F. oxysporum;* (4) IR-displaying sample: seeds were germinated in FS-94 metabolites and further infected with the pathogenic strain 137 of *F. oxysporum*.

RNA isolation, purification, cDNA library construction, sequencing on Genome Analyzer *IIx* (Illumina, USA), and data processing were described elsewhere [32]. Sequencing data are deposited in NCBI at the accession numbers SRR7511483, SRR7511484, SRR7511485 and SRR7511486.

### 4.2. Identification and Characterization of nsLTPs in Wheat Transcriptomes

The pipelines developed in Perl and used for nsLTP identification were the same as for DEFLs described earlier [32]. The first pipeline was based on the method of hidden Markov models. The ready models of LTP precursors were obtained from SPADA [61]. The pipeline works in several steps. First, the hidden Markov models were aligned against the transcriptome with hmm-search from HMMER package [62]. Amino acid sequences in FASTA-like formats were required as input. Second, the discovered sequences were filtered by the Perl scripts. The first script filtered the hits by E-value (E-value < 10^−3^). The second script detected signal peptides in the remaining sequences of LTP precursors using the console version of SignalP v4.1 [63]. Sequences without signal peptides were discarded. The third script checked the discovered sequences to match the structure MZ..Z{C}m{X}n{C}l{X}k..*, where MZ..Z is a signal peptide; M, methionine; Z, any amino acid; C, cysteine; X, any amino acid residue except cysteine; m, n, l, k = 1, 2, 3…; *is a stop codon. Thus, the resulting precursor of a LTP peptide consisted of a signal peptide that started with methionine and a cysteine motif. Sequences with mature peptide length longer than 204 amino acid residues were discarded. After all quality control processes, the nucleotide sequences of peptides were detected by a specific script written in Perl. As a result, a collection of predicted amino acid and nucleotide sequences of identified LTP was generated.

The second pipeline used the method of regular expressions to detect sequences of putative nsLTPs precursors. This pipeline consisted of scripts that scanned transcriptome for sequences that match certain regular expressions. The general structure of regular expressions was shown above. The structure of regular expressions also considered the methionine residue at the beginning of the sequence. After obtaining a set of sequences that satisfied the structure of constructed regular expressions, the identified sequences were filtered by the presence of a signal peptide. At this step, the script from the first pipeline with the corresponding function was used. Finally, the nucleotide sequences were obtained.

The resulting array of sequences was compared to the one obtained by hidden Markov models, and the redundant sequences were excluded.

In all identified putative LTPs, the location of a signal peptide was predicted by SignalP v4.1 [63]. The C-terminal GPI-anchored signals were predicted by the big-PI PPlant Predictor program [64]. Isoelectric point (pI) for each putative mature LTP was calculated by IPC tool [65]. Molecular weight was calculated by ProtParam [66]. For domain identification and GO term prediction, InterProScan was used [67]. The three-dimensional structures of selected LTPs were obtained by Phyre2 and PyMOL [68]. Annotation of putative nsLTPs was carried out by BLAST. Subcellular localization of predicted LTPs was analyzed by TargetP1.1 [69]. All identified putative LTPs were tested with the CS-AMPPred program to predict if they belong to antimicrobial peptides [70]. All alignments were constructed using Vector NTI Advance 9 software. Sequence Logo plots of aligned ECM sequences were generated using WebLogo [71]. Phylogenetic trees based on multiple alignment of mature protein sequences were built by MEGA7 using the Neighbor-joining method [72]. Each node was calculated using 10,000 repeated bootstrap tests. *A. thaliana* nsLTP sequences were retrieved from TAIR databases (www.arabidopsis.org).

### 4.3. Expression Analysis of nsLTP Genes

RNA-seq data obtained earlier were used for expression profiling of nsLTP genes in wheat seedlings treated with the pathogenic *F. oxysporum* strain, the FS-94 elicitors and in seedlings pretreated with the elicitors and infected by the pathogenic fungus. Differential TkLTP gene expression analysis was based on read counts from infected, elicitor-treated, and pretreated with the elicitor and infected seedlings compared to those obtained from untreated control seedlings. To estimate TkLTP gene expression levels, reads from four libraries were mapped to the final assembly produced by combining all libraries using bowtie2 software with default parameters. Raw read counts were obtained by samtools idxstats [73]. Transcript abundance for individual TkLTP coding sequences was calculated as counts per million mapped reads (CPM). Minimal expression threshold was defined as the minimal value of the maximal CPM value of predicted TkLTP in four libraries. Differentially expressed genes were those with an expression fold change ≥2 (upregulation) or ≤0.5 (downregulation).

TkLTP gene expression patterns were represented by heatmaps (R package gplots v3.0.1).

### 4.4. RT-PCR Validation

Three μg of total RNA obtained by combining RNA preparations from all four samples were used for rapid amplification of cDNA ends using the Mint kit (Evrogen, Russia) according to the manufacturer’s instructions. The amplified cDNAs coding specific TkLTPs were synthesized using high-fidelity Tersus DNA polymerase (Evrogen, Russia) and gene-specific primers (Table S3). PCR conditions were as follows: initial denaturation step at 94 °C for 2 min followed by 35 cycles of denaturation at 94 °C for 30 s, primer annealing at 59–63 °C for 30 s, and primer extension at 72 °C for 30 s, with the final extension of 5 min at 72 °C. The amplified fragments were separated by agarose gel electrophoresis and isolated from the gel with the Cleanup Standard kit (Evrogen, Russia). PCR fragments were cloned in pAL2-T vector (Evrogen, Russia). The resulting constructs were sequenced using ABI PRISM 3730 instrument (Applied Biosystems, USA).

### 4.5. Real-Time PCR Analysis

To confirm the expression levels of selected nsLTP genes obtained by RNA-seq, qRT-PCR was used. The list of primers used in PCR is shown in Table S8. qRT-PCR was carried out with the qPCRmix-HS SYBR+HighROX kit (Eurogen, Russia) according to the manufacturer’s protocol on a DT-96 instrument (DNA technology, Russia). The house-keeping genes *ef-1a* (KX533924.1) and *rli* (AY059462.1) were employed as internal reference genes.

PCR conditions were as follows: initial denaturation step at 94 °C for 2 min followed by 40 cycles of denaturation at 94 °C for 30 s, primer annealing at 62–66 °C for 30 s, and primer extension at 72 °C for 30 s, with the final extension of 5 min at 72 °C. The melting curves were generated at 95 °C after the reaction had been terminated.

Each experiment was run in triplicate, in three technical replicates. The relative expression levels were calculated based on the *rli* gene. The fold changes in gene expression were estimated in terms of threshold cycles using the 2^−∆∆C^_T_ method [74]. The PCR amplification specificities of genes were confirmed by sequencing the PCR fragment. The results are presented as the mean ± standard deviation (SD).

## 5. Conclusions

In conclusion, we discovered 243 putative nsLTPs in *T. kiharae*, a highly pathogen-resistant hexaploid wheat species, by global transcriptome sequencing. 121 TkLTP genes including paralogs with identical mature peptides showed differential expression pattern in response to *F. oxysporum* infection, *F. sambucinum* elicitors and upon infection of elicitor-pretreated plants. Variation in expression profiles between duplicated TkLTP genes points to diversification of physiological functions between the paralogs. It is worth noting that another AMP family, DEFLs, displayed a contrasting expression pattern in response to infection of untreated and elicitor-pretreated plants: more DEFL genes were downregulated than upregulated in *F. oxyspoum*-infected plants, while more DEFLs were upregulated than downregulated in IR-expressing plants [32]. Thus, the role of two AMP families—nsLTPs and DEFLs—in response to *F. oxysporum* infection and FS-94 elicitors seems to be different. We speculate that in wheat upregulated TkLTP genes together with other defense genes are involved in activation of defense response to the pathogen either directly due to the antimicrobial or lipid-transferring activity or indirectly as signaling molecules. Among the upregulated genes there might be those providing resistance to *Fusarium* trichothecene mycotoxins, as was shown for two *A. thaliana* LTPs [60]. After in-depth analysis of functions the upregulated TkLTPs may find practical application in control of root rot diseases caused by *Fusarium* species. Another interesting finding in our work is that in IR-displaying plants, the vast majority of nsLTP genes were downregulated suggesting the role of negative regulators of the immune processes for at least some of them. Further detailed studies will elucidate the molecular mechanisms involved.

## Figures and Tables

**Table 1:** Characteristics of different types of *T. kiharae* non-specific lipid-transfer proteins (nsLTPs).

Type	Number of Members	GPI-anchor	Cysteine Spacing Pattern										
1	59	No	C	X9	C	X14,15	CC	X19	CXC	X19,21,23	C	X13,14	C
2	38	No	C	X7	C	X13	CC	X8,9	CXC	X23	C	X6	C
D	43	No	C	X6,9,10,14	C	X13,14,16–18	CC	X9,11–14	CXC	X22-26	C	X7–10	C
G	91	Yes	C	X6,9,10	C	X12–18	CC	X12,14	CXC	X22,27–29	C	X6,8,9	C
X	12	No	C	X8,9	C	X14,16,19,21	CC	X9,12,13	CXC	X18,24,29	C	X6,9	C

## Supplementary Materials

The following materials are available online at https://www.mdpi.com/2076-0817/8/4/221/s1, Figure S1: Multiple sequence alignment of *T. kiharae* nsLTPs, Figure S2: Sequence Logo plots of aligned *T. kiharae* DIR1 mature peptide sequences, Table S1: nsLTP number in plants of different families, Table S2: Types of T. kiharae LTPs, Table S3: List of primers for RT-PCR validation, Table S4: Database accession numbers of *A.thaliana* nsLTPs used in this work, Table S5: Expression patterns of T. kiharae LTP genes, Table S6: TkLTP genes responsive to the elicitors of *F. sambucinum*, *F. oxysporum* infection and to *F. oxysporum* infection after elicitor treatment, Table S7: Up- and downregulated TkLTP genes in IR-expressing *T. kiharae* seedlings compared with *F. sambucinum*-treated and *F. oxysporum*-infected seedlings, Table S8: List of primers for real-time PCR analysis.
