# Supplementary material for: Non-Specific Lipid Transfer Proteins in Triticum kiharae Dorof. et Migush.: Identification, Characterization and Expression Profiling in Response to Pathogens and Resistance Inducers
Source: Pathogens. 2019 Nov 5;8(4):221. doi: 10.3390/pathogens8040221 (PMC6963497; doi:10.3390/pathogens8040221)
Supplement: Supplementary file 1 [file pathogens-08-00221-s001.zip › Table S7.docx]

**Table S7.** Up- and down-regulated TkLTP genes in IR-expressing *T. kiharae* seedlings compared with *F. sambucinum*-treated and *F. oxysporum*-infected seedlings^(1)^

| **IR/Ind*** | | **IR/Inf**** | |
| --- | --- | --- | --- |
| **Up-regulated** | **Down-regulated** | **Up-regulated** | **Down-regulated** |
| TkLTP1.43 | TkLTP1.2 | TkLTP1.43 | TkLTP1.9 |
| TkLTP1.44 | TkLTP1.3 | TkLTP1.44 | TkLTP1.10 |
| TkLTPd9.1 | TkLTP1.4 | TkLTP2.12 | TkLTP1.11 |
| TkLTPd9.2 | TkLTP1.5 | TkLTP2.13 | TkLTP1.12 |
| TkLTPd9.3 | TkLTP1.7 | TkLTPd7.3 | TkLTP1.13 |
| TkLTPd9.4 | TkLTP1.8 | TkLTPg9.1 | TkLTP1.14 |
| TkLTPd9.5 | TkLTP1.10 | TkLTPx2.1 | TkLTP1.15 |
| TkLTPg8.6 | TkLTP1.11 |  | TkLTP1.16 |
| TkLTPg8.7 | TkLTP1.12 |  | TkLTP1.17 |
|  | TkLTP1.13 |  | TkLTP1.18 |
|  | TkLTP1.14 |  | TkLTP1.19 |
|  | TkLTP1.15 |  | TkLTP1.20 |
|  | TkLTP1.16 |  | TkLTP1.21 |
|  | TkLTP1.22 |  | TkLTP1.22 |
|  | TkLTP1.23 |  | TkLTP1.23 |
|  | TkLTP1.24 |  | TkLTP1.24 |
|  | TkLTP1.35 |  | TkLTP1.35 |
|  | TkLTP1.36 |  | TkLTP1.36 |
|  | TkLTP1.37 |  | TkLTP1.37 |
|  | TkLTP1.38 |  | TkLTP1.38 |
|  | TkLTP1.39 |  | TkLTP1.39 |
|  | TkLTP1.40 |  | TkLTP1.40 |
|  | TkLTP1.41 |  | TkLTP1.41 |
|  | TkLTP1.42 |  | TkLTP1.42 |
|  | TkLTP2.25 |  | TkLTP2.8 |
|  | TkLTP2.26 |  | TkLTP2.25 |
|  | TkLTP2.27 |  | TkLTP2.26 |
|  | TkLTP2.28 |  | TkLTP2.27 |
|  | TkLTP2.29 |  | TkLTP2.29 |
|  | TkLTP2.30 |  | TkLTP2.31 |
|  | TkLTP2.31 |  | TkLTP2.32 |
|  | TkLTP2.32 |  | TkLTP2.33 |
|  | TkLTP2.33 |  | TkLTP2.34 |
|  | TkLTP2.34 |  | TkLTP2.35 |
|  | TkLTP2.35 |  | TkLTP2.36 |
|  | TkLTP2.36 |  | TkLTP2.37 |
|  | TkLTP2.37 |  | TkLTP2.38 |
|  | TkLTP2.38 |  | TkLTPd2.4 |
|  | TkLTPd1.1 |  | TkLTPd3.1 |
|  | TkLTPd2.4 |  | TkLTPd3.2 |
|  | TkLTPd3.1 |  | TkLTPd10.1 |
|  | TkLTPd3.2 |  | TkLTPg1.5 |
|  | TkLTPd10.1 |  | TkLTPg1.6 |
|  | TkLTPd11.3 |  | TkLTPg1.7 |
|  | TkLTPd11.4 |  | TkLTPg1.8 |
|  | TkLTPd11.5 |  | TkLTPg12.1 |
|  | TkLTPg1.1 |  | TkLTPg12.2 |
|  | TkLTPg1.4 |  | TkLTPg12.3 |
|  | TkLTPg1.5 |  | TkLTPg12.4 |
|  | TkLTPg1.6 |  | TkLTPg12.5 |
|  | TkLTPg1.7 |  | TkLTPg12.6 |
|  | TkLTPg1.8 |  |  |
|  | TkLTPg1.9 |  |  |
|  | TkLTPg1.10 |  |  |
|  | TkLTPg1.11 |  |  |
|  | TkLTPg5.1 |  |  |
|  | TkLTPg8.8 |  |  |
|  | TkLTPg8.9 |  |  |
|  | TkLTPg8.10 |  |  |
|  | TkLTPg9.1 |  |  |
|  | TkLTPg12.1 |  |  |
|  | TkLTPg12.2 |  |  |
|  | TkLTPg12.3 |  |  |
|  | TkLTPg12.4 |  |  |
|  | TkLTPg12.5 |  |  |
|  | TkLTPg12.6 |  |  |
|  | TkLTPx1.3 |  |  |

^(1)^ Up-regulated LTP genes are those with an expression fold change ≥2, down-regulated LTP genes are those with an expression fold change and ≤0.5. *Differentially expressed LTP genes in IR-expressing compared with elicitor-treated seedlings; ** Differentially expressed LTP genes in IR-expressing compared with *F. oxysporum*-infected seedlings. LTP genes up-regulated in both variants are highlighted yellow. LTP genes down-regulated in both variants are highlighted green.
