# Supplementary material for: Non-Specific Lipid Transfer Proteins in Triticum kiharae Dorof. et Migush.: Identification, Characterization and Expression Profiling in Response to Pathogens and Resistance Inducers
Source: Pathogens. 2019 Nov 5;8(4):221. doi: 10.3390/pathogens8040221 (PMC6963497; doi:10.3390/pathogens8040221)
Supplement: Supplementary file 1 [file pathogens-08-00221-s001.zip › Table S8.docx]

**Table S8.** List of primers for Real-Time PCR analysis.

| № | TkLTP and reference genes | Pr_dir 5' –> 3' | Pr_rev 5' –> 3' | Length (bp) | T primer annealing (℃) |
| --- | --- | --- | --- | --- | --- |
| 1 | TkLTP1.3 | CCCACAGAGCAAGCACACTA | AGTTCGCAACCGCACCCTCC | 137 | 64 |
| 2 | TkLTP1.5 |  | AGTTCGCAACCGCACCCTCG | 137 | 64 |
| 3 | TkLTP1.9 | ATTTAAGCCCATGCATCCCC | GCATGGACGCCACCATGACA | 170 | 66 |
| 4 | TkLTP1.10 |  | GCATGGACGCCACCATGACG | 170 | 66 |
| 5 | TKLTP1.22 | CGCCCTGGTGGCGGCG | CAATCTCCTGACGCCGCTG | 152 | 66 |
| 6 | TkLTP1.24 | GCTACTAAGCTCGTGCTGGC |  | 170 | 66 |
| 7 | TKLTP1.33 | ATGGCTCGCGTGGCACTGC | CGTATGACATGCACGGCG | 124 | 66 |
| 8 | TKLTP1.34 | ATGGCTCGCGTGGCACTGG |  | 124 | 66 |
| 9 | TKLTP1.37 | GTGAGTGAACTGCCAACTCC | GTAGGTCAGGCACATCACA | 70 | 63 |
| 10 | TKLTP1.38 | GTGTCTATGTCTGTACCTTT |  | 147 | 63 |
| 11 | TKLTP1.41 | TGCTCATACGATACTGATGA |  | 239 | 63 |
| 12 | TKLTP1.42 | CCCTTCCGACGTGCATGCAA |  | 290 | 63 |
| 13 | TKLTP2.21 | AGGGGGAGGCGCCATGAAGA | GTACTGCTTCAGGTTGGGGTC | 241 | 64 |
| 14 | TKLTP2.23 | ACGCCATGAAGTACTTGGCC |  | 203 | 64 |
| 15 | TKLTP2.24 | TGGCGCTCCTGGCCTTCTG |  | 224 | 64 |
| 16 | TKLTPd5.5 | GGTGAGGCGACGATGTCGAC | AGCTTCAGCGCCATCTTGT | 143 | 60 |
| 17 | TKLTPd5.6 | GGTGAGGCGACGATGTCGAT |  | 143 | 60 |
| 18 | TKLTPd7.1 | GTAGCACCGACCGATCGAGC | TGAGGACGACCATGAGCGCC | 127 | 66 |
| 19 | TKLTPd7.2 |  | CGACGACGAGGACGACCGTG | 121 | 66 |
| 20 | TKLTPd7.3 |  | GAGGGCGACGAGGAGGAG | 132 | 66 |
| 21 | TKLTPg1.7 | TCGTGGCGGCGGCGGCGTT | TCGTCGCGGTCCTTGACCA | 214 | 64 |
| 22 | TKLTPg1.8 | TGCACTGTTGATGACGCTC |  | 231 | 64 |
| 23 | TKLTPg1.10 | GCTAGTAGTTAGCAGGTT |  | 280 | 64 |
| 24 | TKLTPg1.11 | CGGACTTCGCGGCGGACC |  | 185 | 64 |
| 25 | TKLTPg6.15 | AGCGGGTGCACGCAGACG | GGGTCTTGTTGACGGTGCCGA | 183 | 62 |
| 26 | TKLTPg6.16 | GGTGCACGCAGACTCTC |  | 185 | 62 |
| 27 | WH-RLI  (AY059462.1) | CGATTCAGAGCAGCGTATTGTTG | AGTTGGTCGGGTCTCTTCTAAATG | 242 | 62 |
| 28 | WH-EF (KX533924.1) | CAGATTGGCAACGGCTACG | CGGACAGCAAAACGACCAAG | 227 | 62 |
