# Supplementary material for: Non-Specific Lipid Transfer Proteins in Triticum kiharae Dorof. et Migush.: Identification, Characterization and Expression Profiling in Response to Pathogens and Resistance Inducers
Source: Pathogens. 2019 Nov 5;8(4):221. doi: 10.3390/pathogens8040221 (PMC6963497; doi:10.3390/pathogens8040221)
Supplement: Supplementary file 1 [file pathogens-08-00221-s001.zip › Table S3.docx]

**Table S3.** List of primers for RT-PCR validation.

| № | TkLTP | Pr_dir 5' –> 3' | Pr_rev 5' –> 3' | Length (bp) | T primer annealing (℃) |
| --- | --- | --- | --- | --- | --- |
| 1 | TkLTP1.3/1.5 | GCCTTCACTCACACACCA | GGACACGAGGTCCCGTCAG | 660 | 62 |
| 2 | TkLTP1.8 | AGAGCAAGCACACTACTAGC | ATCTGAAATGGTTCTGATCT | 419 | 62 |
| 3 | TkLTP1.9/1.10 | AATCACTTAGCAAATCTAGC | GGTTCTCCTCAAAGGTAGT | 631 | 63 |
| 4 | TkLTP1.12 | ACTCGATAGGGATGGCCCGC | TTAGCGAATAGTAGAGCAGT | 439 | 62 |
| 5 | TkLTP1.30 | GAAGCACTAGATCCTCGATG | TAACTTAGTGAAGGTTATTG | 364 | 60 |
| 6 | TkLTP1.22/1.24 | AGCAAATCTAGCTATCCCAC | GTCTCTCTCACATGGAGATC | 580 | 62 |
| 7 | TkLTP1.33/1.34 | AGCGCCACCACCACACAAGC | TGATAACAGTGAGTGTCATA | 643 | 60 |
| 8 | TkLTP1.41/1.42 | CTCAACAGCAAGGCTGTGGT | GGAAGGGTTATATATTGAAC | 370 | 60 |
| 9 | TkLTP1.55 | CAGCGCAACCACCATGACGC | AAGCCGATTTCTCAATCAGT | 414 | 62 |
| 10 | TkLTP2.9 | ACCATGGCGGCGTCGAAGGC | TCAGCACCTTGGCAGGCGCA | 301 | 64 |
| 11 | TkLTP2.20 | AGAGTTTCAGAGACGCCATG | TATATGCGTCAGTTTAGCAT | 319 | 60 |
| 12 | TkLTP2.21/2.23/2.4 | CGCCACAACAATCATTGTAG | TTAATTAACCTCGAAGTAG | 704 | 59 |
| 13 | TkLTP2.31 | GCGTGAGCCATGGCGTCCATC | CTAGCAGTGTGGCAGGGCGA | 301 | 63 |
| 14 | TkLTPd3.1/3.2 | AGCATCGGCGTAAAGGGAAC | TGTTCTTGCTTCAGACCTTCG | 396 | 62 |
| 15 | TkLTPd4.1 | AGGCCGTCGCCGCGGCTATG | TATCATTTCGTACCTGACGC | 454 | 63 |
| 16 | TkLTPd5.3 | AGATGGCAATGAGGCCGCTC | CATCAAGGCAGCGTGTAATCT | 398 | 62 |
| 17 | TkLTPd5.5/5.6 | GGTGAGGCGACGATGTCG | CCAAGCCTCATGGCAGGGTG | 377 | 60 |
| 18 | TkLTPd6.3 | ATGGCCAGGTCACAGGCATT | CTGATCATCACGAGCAGTTG | 325 | 62 |
| 19 | TkLTPd7.1/7.2/7.3 | GAGGCGCCATGAAGAACTTG | ATTCAGAATTTCAGATACAG | 440 | 62 |
| 20 | TkLTPd11.3 | CACTCGGCAGCAATGGCAGG | CGCCTTGCTAGATCGCTTAGC | 419 | 62 |
| 21 | TkLTPg1.6 | AGTCGCCACACGAGTGTGAC | AGACAAGCCTATCATCACGCG | 682 | 63 |
| 22 | TkLTPg1.7/1.8 | CGAAAGAGTCGCCACACGAG | CCAACACAATCATGAAAATGC | 673 | 64 |
| 23 | TkLTPg1.10/1.11 | ATGGCAGCTCGGCGTAGT | TCATGCGAGAACAAGGA | 582 | 64 |
| 24 | TkLTPg6.15/6.16 | AGAGGAGCAAGAAATGGCGG | CAATCAGATCAGATCACATG | 550 | 63 |
| 25 | TKLTPx1.1 | TAGCCAGCCGGCGATCGAC | TGGGGTAAAACAACAGTACG | 711 | 61 |
| 26 | TkLTPx2.1 | GTAGCGGTGGTGGGATCATT | AATAGTATCACCGGGATTAG | 460 | 63 |
