# Supplementary material for: Non-Specific Lipid Transfer Proteins in Triticum kiharae Dorof. et Migush.: Identification, Characterization and Expression Profiling in Response to Pathogens and Resistance Inducers
Source: Pathogens. 2019 Nov 5;8(4):221. doi: 10.3390/pathogens8040221 (PMC6963497; doi:10.3390/pathogens8040221)
Supplement: Supplementary file 1 [file pathogens-08-00221-s001.zip › Table S4.docx]

**Table S4.** Database accession numbers of *A.thaliana* nsLTPs used in this work.

| **Name** | **Locus** |
| --- | --- |
| AtLTP1.1 | At2g15050.2 |
| AtLTP1.2 | At2g15325.1 |
| AtLTP1.3 | At2g18370.1 |
| AtLTP1.4 | At2g38530.1 |
| AtLTP1.5 | At2g38540.1 |
| AtLTP1.6 | At3g08770.1 |
| AtLTP1.7 | At3g51590.1 |
| AtLTP1.8 | At3g51600.1 |
| AtLTP1.9 | At4g33355.1 |
| AtLTP1.10 | At5g01870.1 |
| AtLTP1.11 | At5g59310.1 |
| AtLTP1.12 | At5g59320.1 |
| AtLTP2.1 | At1g43665 |
| AtLTP2.2 | At1g43666.1 |
| AtLTP2.3 | At1g43667.1 |
| AtLTP2.4 | At1g48750.1 |
| AtLTP2.5 | At1g66850.1 |
| AtLTP2.6 | At1g73780.1 |
| AtLTP2.7 | At2g14846.1 |
| AtLTP2.9 | At3g18280.1 |
| AtLTP2.10 | At3g57310.1 |
| AtLTP2.11 | At5g38160.1 |
| AtLTP2.12 | At5g38170.1 |
| AtLTP2.13 | At5g38180.1 |
| AtLTP2.14 | At5g38195.1 |
| AtLTPd1 | At5g48485.1 |
| AtLTPd2 | At5g48490.1 |
| AtLTPd3 | At5g55410.1 |
| AtLTPd4 | At5g55450.1 |
| AtLTPd5 | At5g55460.1 |
| AtLTPd6 | At2g37870.1 |
| AtLTPd7 | At3g53980.1 |
| **Name** | **Locus** |
| AtLTPd9 | At1g32280.1 |
| AtLTPd10 | At4g30880.1 |
| AtLTPd11 | At4g33550 |
| AtLTPd12 | At5g56480.1 |
| AtLTPg1 | At1g03103 |
| AtLTPg2 | At1g05450 |
| AtLTPg3 | At1g18280 |
| AtLTPg4 | At1g27950 |
| AtLTPg5 | At1g36150 |
| AtLTPg7 | At1g62790 |
| AtLTPg8 | At1g73550 |
| AtLTPg9 | At1g73560 |
| AtLTPg10 | At1g73890 |
| AtLTPg11 | At2g13820 |
| AtLTPg12 | At2g27130 |
| AtLTPg13 | At2g44290 |
| AtLTPg14 | At2g44300 |
| AtLTPg15 | At2g48130 |
| AtLTPg16 | At2g48140 |
| AtLTPg19 | At3g22600 |
| AtLTPg20 | At3g22620 |
| AtLTPg21 | At3g43720 |
| AtLTPg22 | At3g58550 |
| AtLTPg23 | At4g08670 |
| AtLTPg24 | At4g12360 |
| AtLTPg25 | At4g14805 |
| AtLTPg26 | At4g14815 |
| AtLTPg27 | At4g22630 |
| AtLTPg28 | At4g22666 |
| AtLTPg30 | At5g13900 |
| AtLTPg31 | At5g64080 |
