# Supplementary material for: Non-Specific Lipid Transfer Proteins in Triticum kiharae Dorof. et Migush.: Identification, Characterization and Expression Profiling in Response to Pathogens and Resistance Inducers
Source: Pathogens. 2019 Nov 5;8(4):221. doi: 10.3390/pathogens8040221 (PMC6963497; doi:10.3390/pathogens8040221)
Supplement: Supplementary file 1 [file pathogens-08-00221-s001.zip › Table S6.docx]

**Table S6.** TkLTP genes responsive to the elicitors of *F. sambucinum*, *F. oxysporum* infection and to *F. oxysporum* infection after elicitor treatment ^(1)^

| **Ind/Cont*** | | **Inf/Cont**** | | **IR/Cont***** | |
| --- | --- | --- | --- | --- | --- |
| **Up-regulated** | **Down-regulated** | **Up-regulated** | **Down-regulated** | **Up-regulated** | **Down-regulated** |
| TkLTP1.2 | TkLTP1.9 | TkLTP1.47 | TkLTPd11.1 | TkLTP1.43 | TkLTP1.9 |
| TkLTP1.3 | TkLTP1.21 | TkLTP1.48 |  | TkLTP1.44 | TkLTP1.10 |
| TkLTP1.4 | TkLTP1.53 | TkLTP1.49 |  | TkLTP1.47 | TkLTP1.11 |
| TkLTP1.5 | TkLTP1.54 | TkLTP1.50 |  | TkLTP1.48 | TkLTP1.12 |
| TkLTP1.6 | TkLTP1.57 | TkLTP1.51 |  | TkLTP1.49 | TkLTP1.13 |
| TkLTP1.7 | TkLTP1.58 | TkLTP1.52 |  | TkLTP1.50 | TkLTP1.14 |
| TkLTP1.8 | TkLTP1.59 | TkLTP2.1 |  | TkLTP1.51 | TkLTP1.15 |
| TkLTP1.43 | TkLTPd9.1 | TkLTP2.7 |  | TkLTP1.52 | TkLTP1.16 |
| TkLTP1.44 | TkLTPd9.2 | TkLTP2.8 |  | TkLTP2.12 | TkLTP1.17 |
| TkLTP1.47 | TkLTPd9.3 | TkLTP2.10 |  | TkLTP2.13 | TkLTP1.18 |
| TkLTP1.48 | TkLTPd9.4 | TkLTP2.25 |  | TkLTPd5.5 | TkLTP1.19 |
| TkLTP1.49 | TkLTPd9.5 | TkLTP2.26 |  | TkLTPd5.6 | TkLTP1.20 |
| TkLTP1.50 | TkLTPd11.1 | TkLTP2.27 |  | TkLTPd5.7 | TkLTP1.21 |
| TkLTP1.51 | TkLTPd11.2 | TkLTP2.28 |  | TkLTPd7.1 | TkLTP1.22 |
| TkLTP1.52 | TkLTPx4.1 | TkLTPd10.1 |  | TkLTPd7.2 | TkLTP1.23 |
| TkLTP2.12 | TkLTPx5.3 | TkLTPg11.5 |  | TkLTPd7.3 | TkLTP1.24 |
| TkLTP2.13 | TkLTPx5.4 | TkLTPg11.7 |  | TkLTPd8.1 | TkLTP1.35 |
| TkLTP2.25 | TkLTPx5.5 | TkLTPg12.1 |  | TkLTPg7.4 | TkLTP1.36 |
| TkLTP2.26 |  | TkLTPg12.2 |  | TkLTPg7.6 | TkLTP1.37 |
| TkLTP2.27 |  | TkLTPg12.3 |  | TkLTPg7.7 | TkLTP1.38 |
| TkLTP2.28 |  | TkLTPg12.4 |  | TkLTPg8.6 | TkLTP1.39 |
| TkLTP2.29 |  | TkLTPg12.5 |  | TkLTPg8.7 | TkLTP1.40 |
| TkLTP2.30 |  | TkLTPg12.6 |  | TkLTPg9.1 | TkLTP1.41 |
| TkLTP2.31 |  | TkLTPx3.1 |  | TkLTPg11.5 | TkLTP1.42 |
| TkLTPd8.1 |  |  |  | TkLTPg11.7 | TkLTP1.57 |
| TkLTPd10.1 |  |  |  | TkLTPx2.1 | TkLTP1.58 |
| TkLTPd11.3 |  |  |  | TkLTPx3.1 | TkLTP1.59 |
| TkLTPd11.4 |  |  |  |  | TkLTP2.25 |
| TkLTPd11.5 |  |  |  |  | TkLTP2.26 |
| TkLTPg5.1 |  |  |  |  | TkLTP2.27 |
| TkLTPg6.9 |  |  |  |  | TkLTP2.32 |
| TkLTPg7.6 |  |  |  |  | TkLTP2.33 |
| TkLTPg7.7 |  |  |  |  | TkLTP2.34 |
| TkLTPg8.8 |  |  |  |  | TkLTP2.35 |
| TkLTPg8.10 |  |  |  |  | TkLTP2.36 |
| TkLTPg9.1 |  |  |  |  | TkLTP2.37 |
| TkLTPg12.1 |  |  |  |  | TkLTP2.38 |
| TkLTPg12.2 |  |  |  |  | TkLTPd3.1 |
| TkLTPg12.3 |  |  |  |  | TkLTPd3.2 |
| TkLTPg12.4 |  |  |  |  | TkLTPd11.1 |
| TkLTPg12.5 |  |  |  |  | TkLTPg1.5 |
| TkLTPg12.6 |  |  |  |  |  |
| TkLTPx2.1 |  |  |  |  |  |
| TkLTPx3.1 |  |  |  |  |  |

^(1^) Differentially expressed genes are those with an expression fold change ≥2 (up-regulation) or ≤0.5 (down-regulation); *TkLTP genes responsive to *F. sambucinum* elicitors (Ind) compared with control seedlings (Cont); **TkLTP genes responsive to *F. oxysporum* infection (Inf) compared with control seedlings (Cont); ***TkLTP genes responsive to *F. oxysporum* infection after elicitor treatment (in IR-expressing seedlings) compared with control seedlings (Cont). TkLTP genes up-regulated in all 3 variants are highlighted yellow, TkLTP genes down-regulated in all 3 variants are highlighted green. TkLTP genes up-regulated only in IR-expressing seedlings (primed by the elicitors) are highlighted blue.
