# Supplementary material for: Non-Specific Lipid Transfer Proteins in Triticum kiharae Dorof. et Migush.: Identification, Characterization and Expression Profiling in Response to Pathogens and Resistance Inducers
Source: Pathogens. 2019 Nov 5;8(4):221. doi: 10.3390/pathogens8040221 (PMC6963497; doi:10.3390/pathogens8040221)
Supplement: Supplementary file 1 [file pathogens-08-00221-s001.zip › Table S1.docx]

| **№** | **Family/Species** | **Number of nsLTPs** | **Reference** |
| --- | --- | --- | --- |
| **Brassicaceae** | | | |
| 1 | *Arabidopsis thaliana* | 49 | [6] |
| 2 | *Brassica oleraceae* | 89 | [11] |
| 3 | *B. rapa* | 63 | [12] |
| **Caryophyllaceae** | | | |
| 4 | *Dianthus caryophyllus* | 4 | [16] |
| 5 | *Silena vulgaris* | 7 | [16] |
| 6 | *Silena latifolia* | 22 | [16] |
| 7 | *Stellaria media* | 31 | [16] |
| **Malvaceae** | | | |
| 8 | *Gossipium hirsutum* | 138 | [18] |
| **Poaceae** | | | |
| 9 | *Hordeum vulgare* | 70 | [7] |
| 10 | *Leymus arenarius* | 51 | [9] |
| 11 | *Triticum aestivum* | 156 | [6] |
| 12 | *T. aestivum* | 461 | [10] |
| 13 | *Oryza sativa* | 52 | [6] |
| 14 | *Sorgum bicolor* | 58 | [8] |
| 15 | *Zea mays* | 63 | [8] |
| **Solanaceae** | | | |
| 16 | *Capsicum annuum* | 19 | [13] |
| 17 | *Nicotiana benthamiana* | 17 | [13] |
| 18 | *N. tabacum* | 30 | [13] |
| 19 | *Petunia hybrida* | 10 | [13] |
| 20 | *Solanum esculentum* | 24 | [13] |
| 21 | *S. esculentum* | 64 | [14] |
| 22 | *S. tuberosum* | 22 | [13] |
| 23 | *S. tuberosum* | 83 | [15] |

**Table S1.** nsLTP number in plants of different families.
