# Supplementary figures and images for: Non-Specific Lipid Transfer Proteins in Triticum kiharae Dorof. et Migush.: Identification, Characterization and Expression Profiling in Response to Pathogens and Resistance Inducers
Source: Pathogens. 2019 Nov 5;8(4):221. doi: 10.3390/pathogens8040221 (PMC6963497; doi:10.3390/pathogens8040221)

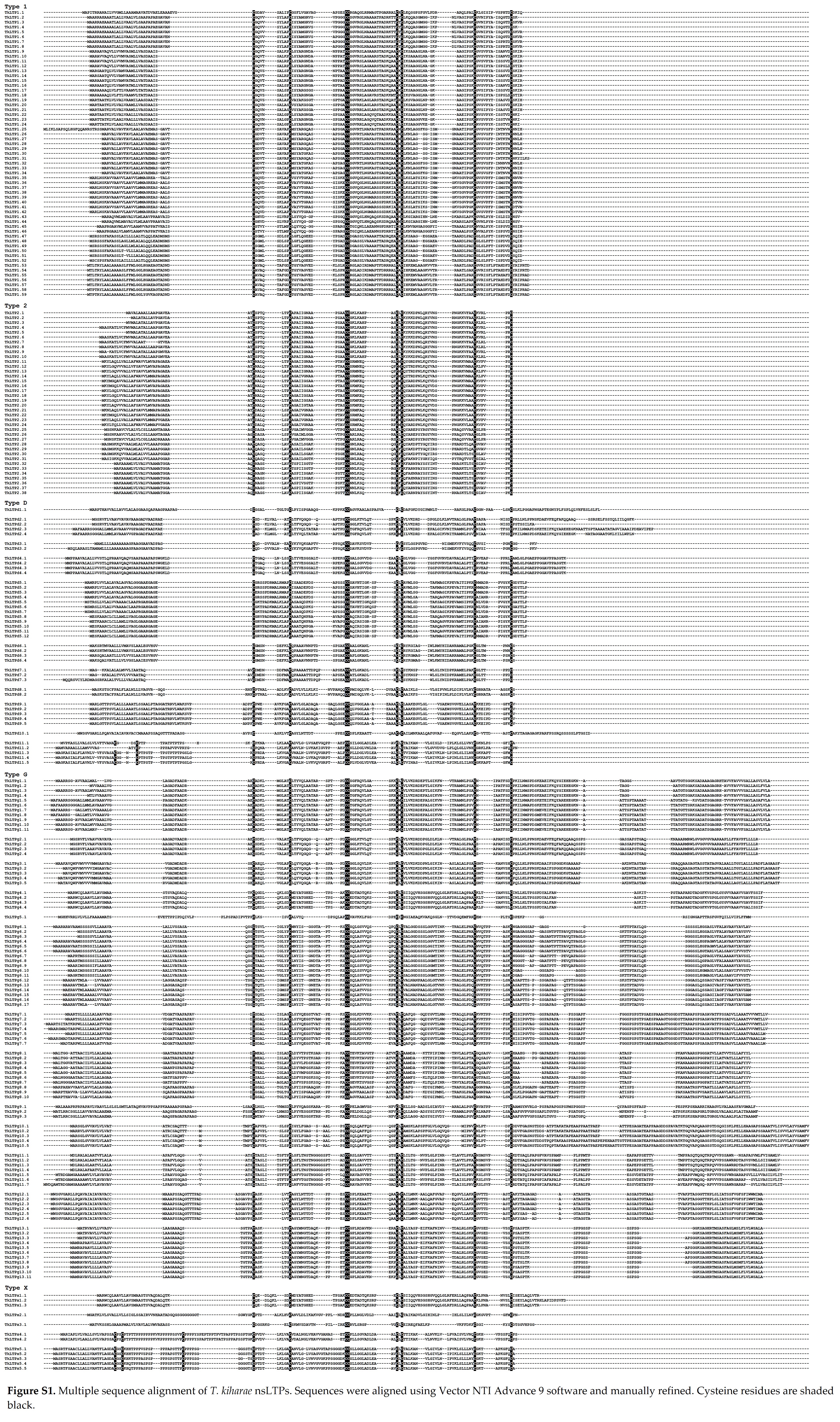

Supplement: Supplementary file 1 [file pathogens-08-00221-s001.zip › Figure S1.jpg]

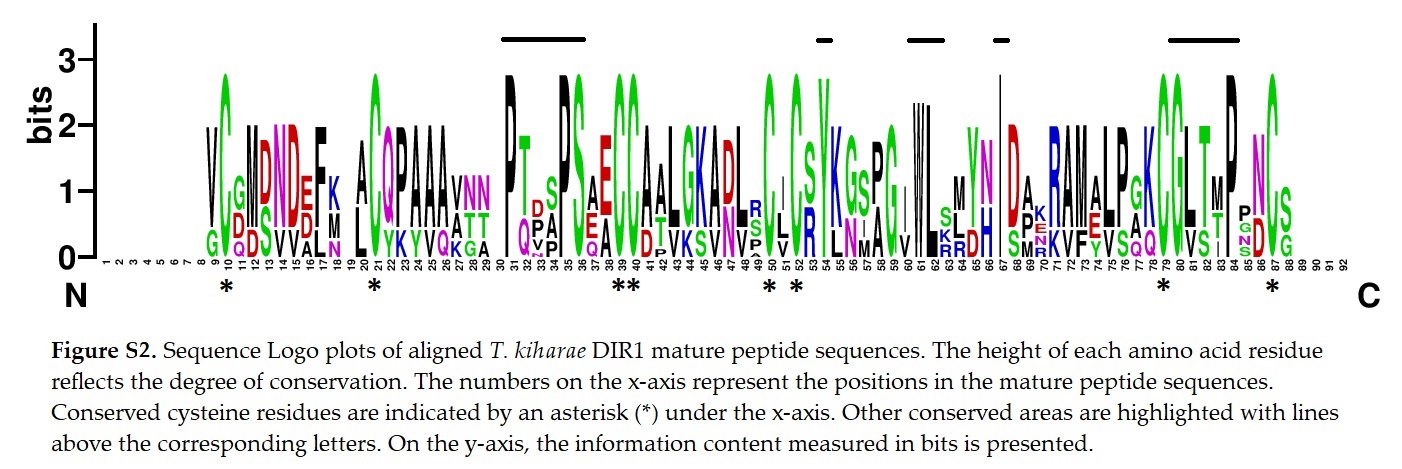

Supplement: Supplementary file 1 [file pathogens-08-00221-s001.zip › Figure S2.jpg]
